# Protein Sequence Comparison Based on Physicochemical Properties and the Position-Feature Energy Matrix

**DOI:** 10.1038/srep46237

**Published:** 2017-04-10

**Authors:** Lulu Yu, Yusen Zhang, Ivan Gutman, Yongtang Shi, Matthias Dehmer

**Affiliations:** 1 School of Mathematics and Statistics, Shandong University at Weihai, Weihai 264209, China; 2 Faculty of Science, University of Kragujevac, P. O. Box 60, 34000 Kragujevac, Serbia; 3 Center for Combinatorics and LPMC, Nankai University, Tianjin 300071, China; 4 Department of Mechatronics and Biomedical Computer Science, UMIT, Hall in Tyrol, Austria; 5 College of Computer and Control Engineering, Nankai University, Tianjin 300071, China

## Abstract

We develop a novel position-feature-based model for protein sequences by employing physicochemical properties of 20 amino acids and the measure of graph energy. The method puts the emphasis on sequence order information and describes local dynamic distributions of sequences, from which one can get a characteristic B-vector. Afterwards, we apply the relative entropy to the sequences representing B-vectors to measure their similarity/dissimilarity. The numerical results obtained in this study show that the proposed methods leads to meaningful results compared with competitors such as Clustal W.

With the rapid development of sequencing technologies, a large number of protein sequences have been generated which increase the bio-scientists’ understanding of organisms. But at the same time, the available information of many sequences has not been defined and, therefore, inferring structure and function of protein sequences effectively became a critical challenge in bioinformatics.

Comparing biological sequences has been an important strategy in molecular biology and bioinformatics and supports other types of analysis, such as prediction of protein sub-cellular localization^1^ and the field of taxonomy^2^. Up to now, numerous approaches have been proposed for comparing biological sequences, which can be subsumed under the apparatus of sequence alignment-based methods^3^,^4^,^5^,^6^ and alignment-free methods. Sequence alignment-based method generally requires to define a sequence alignment scoring matrix and gap penalty parameters to represent the change of letters in the compared structures. But the required computational effort to process large databases may create additional problems when analyzing more complex models to improve our understanding of evolution. Therefore, the research of alignment-free methods based on using quantitative characterization of protein sequences will become necessary and important as it reduces the running times^7^,^8^,^9^,^10^.

Amino acid composition (AAC)^11^ is the simplest alignment-free model representing protein sequences based on *k*-word frequency. But the AAC model does not contain information of sequence-order. In order to overcome this limitation, the powerful pseudo amino acid composition (PseAAC) due to Chou^12^ has been widely applied to various biomedical areas and computational proteomics^13^,^14^. Graphical representation of protein sequences is one of the widely used alignment-free methods, that provide a simple visual model for recognizing mass characteristics among similar biological sequences^15^,^16^,^17^,^18^,^19^. While developing graphical representations and models has been challenging^19^, many researchers explore methods based on characteristic vectors encoding amino acids, assuming that all letters in the sequence are equal and neglecting the importance of local interactions among the neighborhood of the amino acids in the sequence.

More and more researchers have already studied physicochemical properties of 20 amino acids, such as hydrophobicity values, isoelectric point, relative molecular mass and ionization equilibrium constant (*pKa* values)^20^,^21^,^22^ when it comes to sequences comparison. The amino acids are building blocks when modeling the protein structure and each amino acid has its own physicochemical properties^18^. Therefore, extracting the features based on the properties of amino acid is essential and reasonable to compare proteins and study their function.

The energy *E*(*G*) of a graph *G* is based on the eigenvalues of the adjacency matrix of *G*. The measure is due to Gutman^23^. Eventually, it attracted much attention in both mathematics and chemistry, and became an important invariant of research in graph theory^20^,^24^,^25^,^26^.

Therefore, we here propose a novel position-feature model of protein sequences based on physicochemical properties of 20 amino acids and graph energy. According to the specific position of amino acids in the sequence, we construct the position-feature matrices consisting of 0 and 1, and map these matrices to bipartite graphs. By computing the energy *E* of each graph, we obtain a characterizing vector E* for the protein sequence. Modifying the vector E*, we get a protein-based characteristic B-vector and apply relative entropy to analyze the similarity/dissimilarity between sequences. Since the characterizing vector depends on the the length of the sequence, for the B-vectors with different lengths, we adopted the subsequence with the smallest distance value and normalize the B-vectors. In order to demonstrate the feasibility and performance of our method, we use the B-vectors to analyze the similarities of 9 ND5, 24 TFs, 27 AFPs proteins and 50 beta-globin proteins.

## Methods

### Extract Order of Amino Acids Based on Isoelectric Point and pKa

Protein sequences are usually composed of 20 amino acids possessing various physicochemical properties. These properties have been essential factors for predicting the function and structure of protein sequences^18^,^27^,^28^,^29^. Thus we extract the order of amino acids based on 2 typical properties of amino acids: isoelectric point (*PI*) and ionization equilibrium constant (*pKa*). The *PI* is the pH at which there is no net charge on a amino acid. The *pKa* is given by the ratio of the concentration product of the ionized ion and concentration of none-ionized molecules. The data set of the amino acids and 2 physicochemical properties are shown in Table S1, see Supplementary Materials.

In this paper, we couple the *PI* value with *pKa* value to describe amino acids by


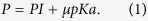


*PI* represents the isoelectric point value of amino acid and *pKa* is the pKa value of amino acid. The parameter *μ* can be used to weight the importance of two physicochemical properties:


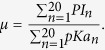


*P* is the integrated value of 2 physicochemical properties.

Finally we obtain the *P* value of each amino acid (presented in Table S1, see Section ‘Supplementary Materials’ and arrange the order of the 20 amino acids in ascending order by calculating the *P* value: *K* → *R* → *A* → *G* → *H* → *W* → *I* → *L* → *V* → *T* → *P* → *S* → *Y* → *Q* → *F* → *M* → *N* → *C* → *E* → *D*.

### Position-Feature Vectors of Protein Sequences

Position-based information of sequences becomes important when comparing sequences. Each amino acid has a specific position in the sequence and local interactions among the neighborhood of the amino acids that are strongly associated with compact structural pattern. Given a biological sequence, in addition to considering the content-based information of it, the position distribution of occurrences of amino acids should be taken into account in sequences comparison. A graph representation can be a suitable mathematical tool to extract the relative position information of amino acids in the sequence. Therefore, based on the graph-energy concept, we construct a position-feature model associated with a (0, 1) matrix to calculate the energy of this network.

### The Energy of a Graph

Let *A* = (*a*_*ij*_) be a 20 × 20 (0, 1)-matrix, that is uniquely associated with a bipartite graph *G*. The rules for drawing this graph *G* are as follows: If we assume that 20 amino acids correspond to 20 points (vertices). The vertices marked by *i* pertain to 20 different amino acids, whereas the vertices marked by *j* correspond to the amino acids in the protein sequence. The graph *G* is a 40-vertex bipartite graph. If *a*_*ij*_ = 1, then there is an edge between vertex *i* and *j*; otherwise, there is no edge between these points.

We can compute all eigenvalues *λ*_1_, *λ*_2_, …, *λ*_*n*_ of the matrix *A* = (*a*_*ij*_) and the energy *G* is defined as refs 20,23, 24, 25, 26,30,31:


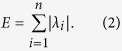


### Obtaining Sparse Matrices and Constructing Characteristic Vectors Based on Protein Sequence

For a protein sequence of length *n*, we design a sliding window of length 20 and shift the sliding window one amino acid at a time from position 1 to *n* − 19. Here, we allow that the sliding window has some overlap within the sequence. For each subsequence of length of 20, we first search for the animo acids in the given subsequence based on the order of amino acids: *K* → *R* → *A* → *G* → *H* → *W* → *I* → *L* → *V* → *T* → *P* → *S* → *Y* → *Q* → *F* → *M* → *N* → *C* → *E* → *D*. The searching rule is defined as follows: Construct a 20 × 20 *D*-matrix, if the *j*-th amino acid in the specific subsequence is just the *i*-th type of amino acid in above order. We define the element in the *i*-th row and the *j*-th column to be 1, otherwise, to be 0.

So, we obtain *n* − 19 sparse matrices and map to *n* − 19 bipartite graphs in the just described way. Finally, we compute the energy *E* of each graph and construct a (*n* − 19)-dimensional characterizing vector E* = (*E*_1_, *E*_2_, …, *E*_*n*−19_) for the protein sequence.

### B-vector: Protein Characteristic Vector

Given a protein sequence of length *n*, we define its B-vector as:


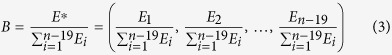


where *E*_*i*_ is defined by equation (2).

Obviously, every component of the B-vector satisfies the following conditions:


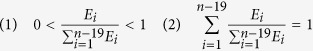


Therefore, the B-vector represents a probability distribution.

For example, we consider a short segment of a protein from yeast Saccharomyces cerevisiae. The length of the sliding window is 20, and it shifts one amino acid at a time. The segment is shown as follows:


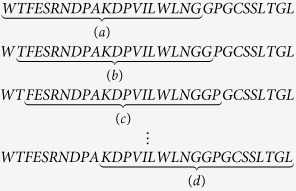


**Figure 1:**
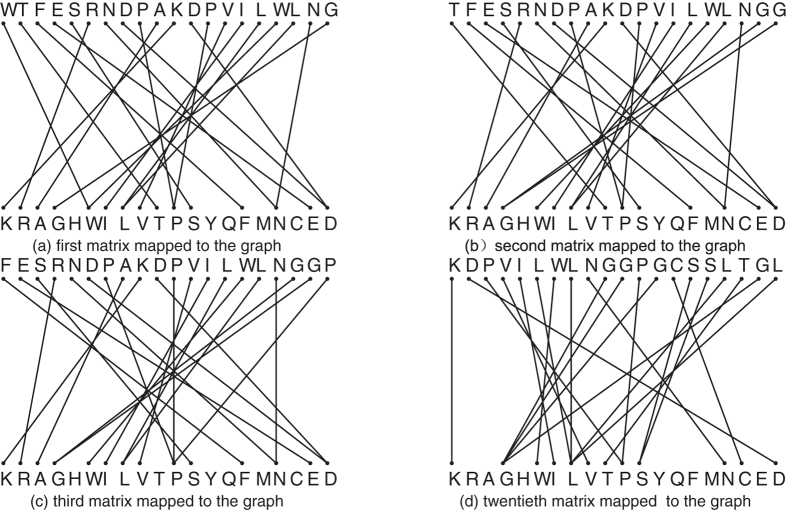
Matrix mapped to the bipartite graph.

Now we only show the fist matrix (a) of this segment. The result is as follows:


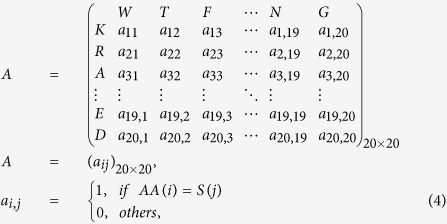


where *AA*(*i*) indicates the *i*-th kind of amino acid in the arranged order whereas *S*(*j*) indicates the *j*-th kind of amino acid from the segment (a). The above matrix which is mapped to the following bipartite graph (a) is shown in Fig. 1.

As shown in Fig. 1, the second matrix is mapped to graph (b) coming from the second sliding window, and the third matrix is mapped to graph (c) coming from the third sliding window. The last one is the graph (d). The length of the above protein segment is 30, so we obtain 11 sparse matrices and 11 bipartite graphs. Then, by computing the energy of every graph, we obtain an 11-dimensional characteristic vector E* = (7, 6, 5, 6, 8, 3, 8, 7, 4, 5, 2) and an 11-dimensional B-vector.

### Numerical Characterization of Protein Sequence

The quantitative comparison of sequences can be done based on traditional distance equations, such as the Euclidean distance, Kullback-Leibler divergence (KLD), Chebyshev distance, and the Manhattan distance. Here, we apply the relative entropy (symmetrical Kullback-Leibler distance^32^,^33^,^34^,^35^,^36^,^37^), as an index for measuring the similarity or distance between two protein sequences based on their B-vectors. The underlying B-vectors capture structural information. Generally, the more similar two protein sequences are, the smaller is the distance value between the sequences.

A similar biological problem compared to the present one was considered in ref. 38. There, Emmert-Streib and Dehmer measured the fragility of genes in a transcriptional regulatory network by modelling its information processing by using a first order Markov chain; afterwards they studied the influence of single gene perturbations on the global and asymptotic communication among the genes, see ref. 38.

### Relative Entropy Distance

If B-vectors of two sequences are denoted by 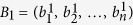 and 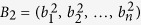, respectively, then the relative entropy between two B-vectors can be calculated by:


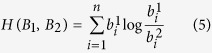



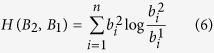



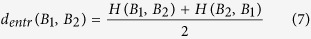


### Normalized B-vectors of Protein Sequence

When the lengths of the compared two sequences are being equal, then the dimensions of their B-vectors are identical. Then we calculate their distance by means of the relative entropy distance. For the protein sequences with different lengths, we construct a slipping window. Assume that the length of protein S1 is *m* and the length of protein S2 is *n (m* > *n*). We take *n* length as a window slipping one step at a time on S1, and obtain *n* − *m* + 1 subsequences. Then we can measure the relative entropy distances between each subsequence and protein S2 respectively, and choose the subsequence with the smallest value representing the S1 to compare with S2.

## Results and Discussion

### Application in the Similarity Analysis of Protein Sequence

Comparison of different biological sequences has been an important way to analyze biological sequences. In order to demonstrate the efficiency of our method, we apply it to some real protein sequence data: 9 ND5, 24 TFs, 27 AFPs proteins and 50 beta-globin proteins. Then, we compare the results of our approach with those of Clustal W and other literatures, which shows that our results are effective and meaning. Note that Clustal W is a widely used multiple-sequence alignment program for DNA or proteins in molecular biology.

### Similarity Analysis of 9 ND5 Protein Sequences

**Figure 2:**
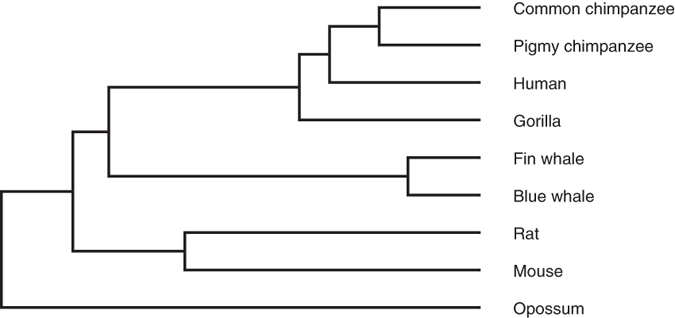
Phylogenetic tree of the 9 ND5 proteins constructed by our method.

Several similarity/dissimilarity methods have been proposed to analyze the nine NADH Dehydrogenase 5 (ND5) protein sequences^18^,^37^,^39^,^40^,^41^. Because of its high mutation rate, ND5 has been widely used for the analysis of the phylogenetic and population genetic diversity of the cats. We apply our method to analyze the similarity of the 9 ND5 proteins whose detailed descriptions in NCBI are shown by Table S2, see Supplementary Materials. Then, we construct the phylogenetic tree depicted in Fig. 2 indicating the evolutionary relationship of 9 species. In order to illustrate the effectiveness of our method, we compare the result of our approach with the ones of Clustal W (see the second figure in ref. 42 for details).

As we can see from the phylogenetic tree that the distance between Fin whale and Blue whale is the smallest, so they are more similar than the four species (Pigmy chimpanzee, Common chimpanzee, Human and Gorilla) and two species (Rat and Mouse). Human - chimpanzee has a relatively closer relationship than Human - Gorilla, which concurs with the known evolutionary facts. Besides, Opossum is the most distant from other eight species, confirming that Opossum is unique among the 9 species. This shows that the result by using our method is completely consistent with the one obtained by Clustal W.

### Similarity Analysis of 24 TFs

The 24 transferrins (TFs) sequences from 24 vertebrates whose detailed information are shown in Table S3, see Supplementary Materials, have been analyzed extensively, see, e.g., refs 2,43,44. All the sequences have been obtained from the NCBI genome database.

**Figure 3:**
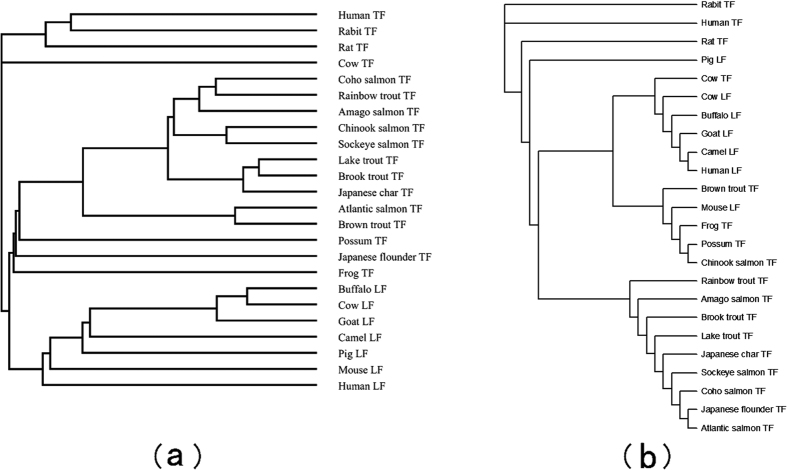
(**a**) Phylogenetic tree of 24 TFs constructed by our method. (**b**) Phylogenetic tree of 24 TFs constructed by Clustal W.

We calculate the relative entropy between two B-vectors of 24 TFs and construct the phylogenetic tree of sequences. The tree can be seen in Fig. 3(a,b) (the ninth figure in ref. 24) illustrates the phylogenetic tree constructed by Clustal W. We find that all these TF proteins and Lactoferrin (LF) proteins could be separated well from Fig. 3(a). In addition, one finds that the tree is almost consistent with the tree constructed by Ford^44^, which is the classical result among the published trees.

### Similarity Analysis of 27 AFPs Proteins

We apply our method to analyze the similarity of 27 Antifreeze proteins (AFPs) which form a set of proteins being able to bind and inhibit the growth of macromolecular ice. These proteins are from spruce budworm (Choristoneura fumiferana, CF), yellow mealworm (Tenebrio molitor, TM), Hypogastrura harveyi(HH), the Dorcus curvidens binodulosus(DCB), Microdera dzhungarica punctipennis(MDP) and Dendroides canadensis(DC)^45^,^46^,^47^,^48^, which all are available in public database. We got the 27 protein sequences from the Supplementary Material *freeze27*.*txt* in ref. 49.

**Figure 4:**
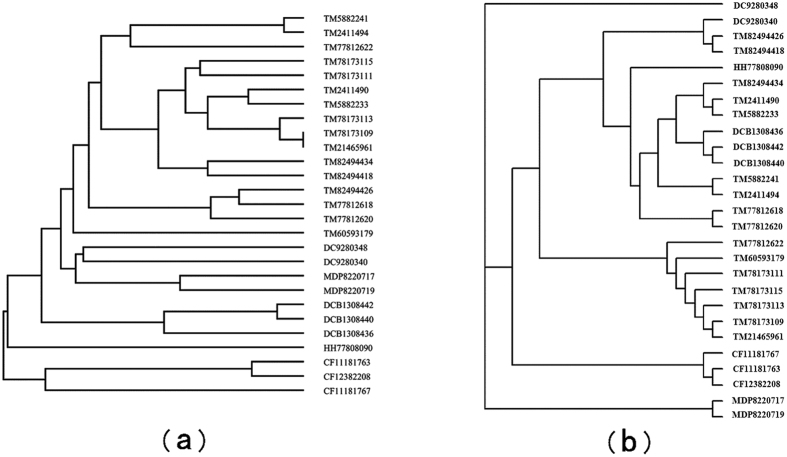
(**a**) Phylogenetic tree of 27 AFPs constructed by our method. (**b**) Phylogenetic tree of 27 AFPs constructed by Clustal W.

In order to indicate the validity of our method, the phylogenetic trees are constructed in Fig. 4(a). As a contrast, the tree constructed by Clustal W is shown in Fig. 4(b) (the twelfth figure in ref. 24). We see that all species are reasonably classified in Fig. 4(a), while in Fig. 4(b), TM proteins are divided into three groups and HH is close to a TM protein. This demonstrates that our outperforms Clustal W.

### Similarity Analysis of 50 Beta-Globin Proteins

**Figure 5:**
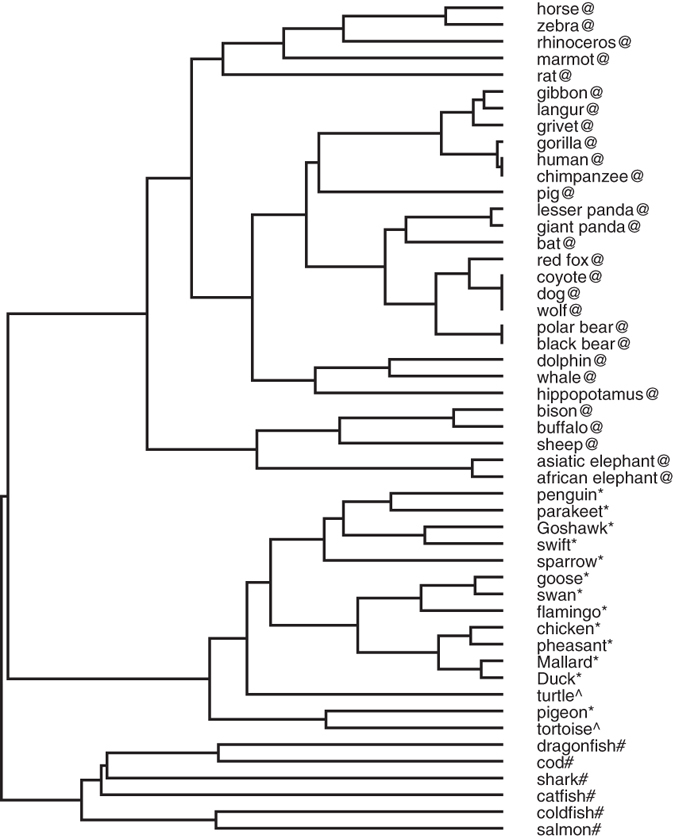
Phylogenetic tree of 50 beta-globin proteins constructed by our method.

50 beta-globin protein sequences of different species^50^ were extracted from GenBank which are shown in Table S4 in the Supplementary Materials. The phylogenetic trees constructed by our method is shown in Fig. 5. We observe that the error rate when it comes to false classification of the species is close to zero and the result of our method is comparably good as the one obtained in ref. 51.

## Conclusion

This paper provides an alignment-free measure, developing a novel position-feature-based model for analyzing protein sequences based on physicochemical properties of 20 amino acids and graph energy. For computing the graph energy, we constructed slipping windows of length 20 to extract the position feature of protein sequence instead of utilizing codes. We transformed each protein sequence of length *n* to *n* − 19 sparse matrices and mapped these matrices to *n* − 19 bipartite graphs. By computing the energy *E* of each graph we got an (*n* − 19)-dimensional characterizing vector E* for each protein sequence. Based on the characterizing vector E*, we obtained a protein-based characteristic B-vector and applied relative entropy (Kullback-Leibler distance^34^) to analyze the similarity/dissimilarity between sequences. Since the characterizing vector depends on the the length of the sequence, for the B-vectors with different lengths, we used for comparative purposed the subsequence with the smallest distance value from the longer one.

Evidenced by numerical results, we have demonstrated that our method measured the similarity/dissimilarit of protein sequences meaningfully and efficiently. Finally, the order of amino acids to construct the matrix have an effect on the sequence comparison, in order to improve the performance of our method, we plan to use different ordering schemas for constructing the matrix to determine the energy of the graph as future work.

## Additional Information

**How to cite this article:** Yu, L. *et al*. Protein Sequence Comparison Based on Physicochemical Properties and the Position-Feature Energy Matrix. *Sci. Rep.* **7**, 46237; doi: 10.1038/srep46237 (2017).

**Publisher's note:** Springer Nature remains neutral with regard to jurisdictional claims in published maps and institutional affiliations.

## Supplementary Material

Supplementary Materials
