# Erratum: Protein Sequence Comparison Based on Physicochemical Properties and the Position-Feature Energy Matrix

**DOI:** 10.1038/srep46787

**Published:** 2017-05-04

**Authors:** Lulu Yu, Yusen Zhang, Ivan Gutman, Yongtang Shi, Matthias Dehmer

Scientific Reports 7: Article number: 4623710.1038/srep46237; published online: 04 10 2017; updated: 05 04 2017

In the original version of this Article, Yusen Zhang was incorrectly affiliated with ‘Faculty of Science, University of Kragujevac, P. O. Box 60, 34000 Kragujevac, Serbia’. The correct affiliation is listed below.

School of Mathematics and Statistics, Shandong University at Weihai, Weihai 264209, China.

This error has now been corrected in the PDF and HTML versions of the Article.

