# Supplementary material for: Protein Sequence Comparison Based on Physicochemical Properties and the Position-Feature Energy Matrix
Source: Sci Rep. 2017 Apr 10;7:46237. doi: 10.1038/srep46237 (PMC5385872; doi:10.1038/srep46237)
Supplement: Supplementary Materials [file srep46237-s1.pdf]

Protein Sequence Comparison Based on Physicochemical Properties and Position-Feature Energy Matrix

Lulu Yu[1], Yusen Zhang[1,*], Ivan Gutman [2], Yongtang Shi[3], Matthias Dehmer[4,5]

[1] *School of Mathematics and Statistics, Shandong University at Weihai, Weihai 264209, China*
[2] *Faculty of Science, University of Kragujevac, P. O. Box 60, 34000 Kragujevac, Serbia.*
[3] *Center for Combinatorics and LPMC, Nankai University, Tianjin 300071, China.*
[4] *Department of Mechatronics and Biomedical Computer Science, UMIT, Hall in Tyrol, Austria*
[5] *College of Computer and Control Engineering, Nankai University, Tianjin 300071, China*
* *Corresponding author: zhangys@sdu.edu.cn*

Supplementary Materials

Table S1: Two typical physicochemical properties value of 20 amino acids.

amino acid	abbreviations	code	PI	pKa	PI+μpKa
Alanine	Ala	A	6	2.4	12.7720
Arginine	Arg	R	10.76	1.8	15.8390
Asparagine	Asp	N	5.41	2.1	11.3355
Aspartate	Asp	D	2.77	2	8.4134
Cysteine	Cys	C	5.07	1.9	10.4312
Glutamine	Gln	Q	5.65	2.2	11.8677
Glutamate	Glu	E	3.22	2.1	9.1455
Glycine	Gly	G	5.97	2.4	12.7420
Histidine	His	H	7.59	1.8	12.6690
Isoleucine	Ile	I	6.02	2.3	12.5099
Leucine	Leu	L	5.98	2.3	12.4699
Lysine	Lys	K	9.74	2.2	15.9477
Methionine	Met	M	5.74	2.1	11.6655
Phenylalanine	Phe	F	5.48	2.2	11.6877
Proline	Pro	P	6.3	2	11.9434
Serine	Ser	S	5.68	2.2	11.8877
Threonine	Thr	T	6.16	2.1	12.0855
Tryptophan	Trp	W	5.89	2.4	12.6620
Tyrosine	Tyr	Y	5.66	2.2	11.8677
Valine	Val	V	5.96	2.2	12.1677

Table S2: The concise information for 9 ND5 protein sequences.

Number	Species	ID(NCBI)	Length
1	Human(Homo sapiens)	AP_000649	603
2	Gorilla(Gorilla gorilla)	NP_008222	603
3	Common chimpanzee(Pan troglodytes)	NP_008196	603
4	Pigmy chimpanzee(Pan paniscus)	NP_008209	603
5	Fin whale(Balenoptera physalus)	NP_006899	606
6	Blue whale(Balenoptera musculus)	NP_007066	606
7	Rat(Rattus norvegicus)	AP-004902_	610
8	Mouse(Mus musculus)	NP_904338	607
9	Opossum (Didelphis virginiana)	NP_007105	602

Table S3: The concise information for 24 TF protein sequences.

Sequence name	Species	Accession no.	Length
Human TF	Homo sapiens	S95936	698
Rabbit TF	Oryctolagus coniculus	X58533	695
Rat TF	Rattus norvegicus	D38380	698
Cow TF	Bos Taurus	U02564	704
Buffalo LF	Bubahts arnee	AJ005203	708
Cow LF	Bos Taurus	X57084	708
Goat LF	Copra hircus	X78902	708
Camel LF	Camehts dromedaries	AJ131674	708
Pig LF	Sus scrofa	M92089	704
Human LF	H. sapiens	NM_002343	710
Mouse LF	Mus musculus	NM_008522	707
Possum TF	Trichosurus vulpecula	AF092510	711
Frog TF	Xenopus laevis	X54530	702
Japanese flounder TF	Pctralichthys olivaceiis	D88801	685
Atlantic salmon TF	Salmo salar	L20313	690
Brown trout TF	Salmo trutta	D89091	691
Lake trout TF	Salvelimts namaycush	D89090	691
Brook trout TF	Sahelinus fontinalis	D89089	691
Japanese char TF	Sahelinus phius	D89088	691
Chinook salmon TF	Oncorhynchus tshawytscha	AH008271	677
Coho salmon TF	Oncorhynchus kisuich	D89084	691
Sockeye salmon TF	Oncorhynchus nerka	D89085	691
Rainbow trout TF	Oncorhynchus mykiss	D89083	691
Amago salmon TF	Oncorhynchus masou	D89086	691

$Note : TF - transferrin;$
$LF - lactoferrin.$

Table S4: 50 beta-globin sequences of animal species.

Animal names	Accession number	Animal names	Accession number
Human	AAA16334.1	Pigeon	P11342.1
Goshawk	P08851.1	Black bear	P68012.1
Lesser panda	P18982.1	Asiatic elephant	P02084.1
Giant panda	P18983.2	African elephant	P02085.1
Sheep	P02075.2	Tortoise	P83123.3
Duck	P02114.2	Grivet	P02028.1
Mallard	P02115.1	Gorilla	P02024.2
Goose	P02117.1	Shark	P02143.1
Rat	CAA33114.1	Hippopotamus	P19016.1
Penguin	P80216.1	Horse	P02062.1
Swift	P15165.1	Gibbon	P02025.1
Coyote	P60525.1	Whale	P18984.1
Catfish	O13163.2	Bat	P24660.1
Bison	P09422.1	Red fox	P21201.1
Swan	P68945.1	Marmot	P08853.1
Buffalo	P67820.1	Salmon	Q91473.3
Dog	P60524.1	Sparrow	P07406.1
Chimpanzee	P68873.2	Pheasant	P02113.1
Dolphin	P18990.1	Flamingo	P02121.1
Goldfish	P02140.1	Pig	P02067.3
Polar bear	P68011.1	Dragonfish	ADD73488.1
Rhinoceros	P09907.1	Parakeet	P21668.1
Chicken	P02112.2	Zebra	P67824.1
Wolf	P60526.1	Cod	O13077.2
Turtle	P13274.1	Langur	P02032.1
